# Blends of Semiflexible Polymers: Interplay of Nematic Order and Phase Separation

**DOI:** 10.3390/polym13142270

**Published:** 2021-07-11

**Authors:** Andrey Milchev, Sergei A. Egorov, Jiarul Midya, Kurt Binder, Arash Nikoubashman

**Affiliations:** 1 Institute for Physical Chemistry, Bulgarian Academia of Sciences, 1113 Sofia, Bulgaria; milchev@ipc.bas.bg; 2 Institute of Physics, Johannes Gutenberg University Mainz, Staudingerweg 7, 55128 Mainz, Germany; sae6z@virginia.edu (S.A.E.); j.midya@fz-juelich.de (J.M.); kurt.binder@uni-mainz.de (K.B.); 3 Department of Chemistry, University of Virginia, Charlottesville, VA 22901, USA; 4 Theoretical Soft Matter and Biophysics, Institute for Advanced Simulation and Institute of Complex Systems, Forschungszentrum Jülich, 52425 Jülich, Germany

**Keywords:** semiflexible polymers, macromolecules, phase behavior, liquid crystals, nematic order, molecular dynamics, density functional theory, mixtures, blends

## Abstract

Mixtures of semiflexible polymers with a mismatch in either their persistence lengths or their contour lengths are studied by Density Functional Theory and Molecular Dynamics simulation. Considering lyotropic solutions under good solvent conditions, the mole fraction and pressure is systematically varied for several cases of bending stiffness κ (the normalized persistence length) and chain length *N*. For binary mixtures with different chain length (i.e., $N_{A}=16$, $N_{B}=32$ or 64) but the same stiffness, isotropic-nematic phase coexistence is studied. For mixtures with the same chain length (N=32) and large stiffness disparity ($\kappa_{B}/\kappa_{A}=4.9$ to 8), both isotropic-nematic and nematic-nematic unmixing occur. It is found that the phase diagrams may exhibit a triple point or a nematic-nematic critical point, and that coexisting phases differ appreciably in their monomer densities. The properties of the two types of chains (nematic order parameters, chain radii, etc.) in the various phases are studied in detail, and predictions on the (anisotropic) critical behavior near the critical point of nematic-nematic unmixing are made.

## 1. Introduction

Semiflexible polymers are macromolecules with linear chemical architecture in the simplest case, which display considerable bending rigidity along the chain backbone [1,2,3,4]. In lyotropic solutions of semiflexible polymers, the competition between orientational and translational entropy can induce a transition from an isotropic (i) to a nematic (n) phase. Depending on the chemical nature of the monomeric units, the persistence length $\ell_{p}$ of such macromolecules can vary over several orders of magnitude: For synthetic polymers, $\ell_{p}$ typically lies in the range from 1nm to 30nm [5], while the distance between repeat units along the backbone, $\ell_{b}$, is typically on the order of several angstroms. Much larger values for these lengths may occur for biologically relevant polymers, e.g., $\ell_{p}\approx 50\;\mathrm{nm}$ for double stranded DNA [6] and $\ell_{p}\approx 17\;\mu m$ for filamentous actin [7]. Note that $\ell_{p}$ does not only depend on the specific polymer chemistry [8] but may also depend on external factors, e.g., the nature of the solvent [6], and the density of monomeric units in a nematically ordered solution or melt [9].

The ability to control the materials properties of polymeric systems is highly desirable for many practical applications. It is common to do this by mixing two chemically different polymers, e.g., blending poly(phenylene ether) resins with polystyrene results in materials with high heat resistance and strong mechanical stability [10]. The statistical thermodynamics of such polymer blends is usually studied via the Flory-Huggins (FH) solution theory [11,12,13,14,15,16], which describes the polymer mixture using a lattice model. Here, the entropy of mixing favors homogeneous systems even for very long chains, whereas unmixing is driven by the chemical incompatibility of the polymers, typically quantified through the FH χ parameter. One central approximation of the FH solution theory is the mean-field description of the energy of mixing, which completely disregards the (local) chain connectivity. As a consequence, this contribution is identical for regular solutions, polymer solutions, and blends [15]. Further, chain stiffness does not enter at all in the standard FH solution theory, so that it cannot distinguish between fully flexible and stiff polymers. Therefore, alternative approaches have been developed to describe rod-coil mixtures, where the flexible coil-like polymer is often modeled as an effective soft spherical particle [17,18,19,20]. Although such coarse-grained descriptions can provide important insights on the (qualitative) phase behavior, they completely lose information on the scale of monomeric units, and are also unsuitable for describing blends of semiflexible polymers, which can neither be described as strictly hard rods nor as soft spheres.

In this work, we consider lyotropic solutions containing two types (A, B) of semiflexible polymers with finite persistence lengths $\ell_{p}^{B}>\ell_{p}^{A}\gg \ell_{b}$. This problem has been previously considered by a few analytical approaches, yielding interesting explicit results only for special limiting cases. Semenov and Subbotin [21] generalized the Onsager-type [22] treatment of single-component lyotropic dilute polymers solution [23,24,25,26] to the two-component case. For the limiting case where the contour lengths $L_{A}$ and $L_{B}$ are in the range $L_{A}\gg \ell_{p}^{A}\gg \ell_{b}$, $L_{B}\gg \ell_{p}^{B}\gg \ell_{b}$, they predicted interesting (qualitative) phase diagrams containing both mixed and coexisting nematic phases [21]. Experimentally, the coexistence of two nematic phases n${}_{1}$ and n${}_{2}$ was observed in mixed virus suspensions [27], which can be modeled theoretically by mixtures of hard rods differing in diameter [28,29]. In recent MD simulations, Zhou et al. studied the behavior of mixtures of semiflexible (ring) polymers in spherical [30] and ellipsoidal [31] containers, finding a confinement-induced phase separation of the two species. In thermotropic systems, n${}_{1}$–n${}_{2}$ coexistence was also predicted for solutions of two kinds of rigid rods with suitable enthalpic interactions [32], and experimental observations of n${}_{1}$–n${}_{2}$ unmixing were also reported for mixtures of side-chain liquid crystalline polymers with small molecule liquid crystals [33], but all such temperature-driven phase transitions are out of consideration here. Thus, we also do not discuss in detail the theory of Liu and Fredrickson [34], which was based on a Landau expansion in terms of both the nematic order parameter and deviations of the local volume fraction of monomeric units from its average. Since Landau expansions require that order parameters are small, this approach is not suitable deeply in the nematic phase. In addition, it was assumed that the transitions were driven by standard enthalpic interactions throughout, i.e., i-i unmixing due to a standard FH χ parameter, and nematic ordering due to a Maier-Saupe-like term [35].

In the present work, we focus on systems where transitions are solely driven by entropic interactions, focusing on cases where contour and persistence lengths are of the same order. Preliminary results of our work were already presented as a letter [36]; in a complementary study [37], we have described related results for shorter chains, with emphasis on a comparison between polymers with finite stiffness and strictly hard rods, and the classification of various contributions to the Gibbs excess free energy within the Density Functional Theory (DFT) framework. In Section 2, we briefly characterize the employed models and methods. Following our previous work on one-component solutions of semiflexible polymers [9,38,39,40], we use both DFT calculations and Molecular Dynamics (MD) simulations for closely related models. Section 3 gives an overview of the possible phase diagrams, emphasizing their description in different statistical ensembles, as predicted by DFT. In Section 4, we analyze in more detail selected systems that were studied by both DFT and MD. For one example, we show how a stiffness mismatch leads to an effective χ parameter of entropic origin, by applying an approach pioneered by Fredrickson et al. [41] and Kozuch et al. [42]. We also discuss the anisotropic character of critical fluctuations for the n-n critical point. Finally, Section 5 summarizes our results and briefly mentions pertinent experiments [43,44].

## 2. Models, Methods, and Phenomenological Concepts

### 2.1. Models and Recorded Observables

In the context of DFT [9,36,37,38,39,40], it is convenient to describe the polymer chains as sequences of *N* tangent hard spheres of diameter σ. Bending stiffness is introduced via a bending potential $U_{\mathrm{bend}}\left(\theta_{ijk}\right)$, which depends on the angle $\theta_{ijk}$ between subsequent bond vectors $a_{i}=r_{i}-r_{j}$ and $a_{j}=r_{k}-r_{j}$, with monomer positions $r_{\mu }$, μ=1,…,N. This potential is chosen as

(1) $$U_{\mathrm{bend}}=\varepsilon_{\mathrm{bend}}\left[1-\cos(\theta_{ijk})\right]\approx \frac{1}{2}\varepsilon_{\mathrm{bend}}\theta_{ijk}^{2},$$

where we anticipated that $\kappa \equiv \varepsilon_{\mathrm{bend}}/\left(k_{B}T\right)\gg 1$, hence small angles $\theta_{ijk}$ dominate. The dimensionless parameter κ controls the persistence length $\ell_{p}$ of the chains, which is traditionally defined from the exponential decay of bond orientational correlations, $\langle a_{i}\cdot a_{i+s}\rangle \propto \exp\left(-s\ell_{b}/\ell_{p}\right)$ [15,45], with bond length $\ell_{b}=\sigma$ here. However, this exponential decay with *s* is doubtful in various contexts [8]; in particular, it does not apply for large *s* when nematic order is present. But even then, the case s=1 can be used to define an effective persistence length $\ell_{p}^{\mathrm{eff}}$:

(2) $$\ell_{p}^{\mathrm{eff}}/\ell_{b}=-1/\ln\langle \cos\left(\theta_{ijk}\right)\rangle \approx 2/\langle \theta_{ijk}^{2}\rangle \;.$$

For simplicity, the superscript “eff” in Equation (2) will be omitted in the following.

The nematic order parameter *S* is defined as the largest eigenvalue of the traceless tensor $Q^{\alpha \beta }\left(\alpha ,\beta =x,y,z\right)$

(3) $$Q^{\alpha \beta }=\frac{1}{2}\left(3\langle u_{i}^{\alpha }u_{i}^{\beta }\rangle -\delta^{\alpha \beta }\right),$$

with unit vector $u_{i}$ along $a_{i}$. The nematic director is defined from the eigenvector belonging to the largest eigenvalue of $Q^{\alpha \beta }$. In a system with a single kind of polymer, the average 〈⋯〉 in Equation (2) is taken over all bond angles of all chains, and over all unit vectors in Equation (3). Correspondingly, in a two-component system with two kinds of polymers A and B (we use $\sigma_{A}=\sigma_{B}=\sigma$ and $\ell_{b}^{A}=\ell_{b}^{B}=\sigma$, but have $\kappa_{A}<\kappa_{B}$), only A chains are included in Equations (2) and (3) for computing $\ell_{p}^{A}$ and $S^{A}$, respectively, and likewise for the B chains.

In the MD simulations, all monomeric units have identical masses $m_{A}=m_{B}=m$. Excluded volume interactions between beads are described by the Weeks-Chandler-Andersen (WCA) potential [46]

(4) $$\begin{array}{c} U_{\mathrm{WCA}}\left(r\right)=\left\{\begin{array}{cc} 4\varepsilon \left[{\left(\frac{\sigma }{r}\right)}^{12}-{\left(\frac{\sigma }{r}\right)}^{6}\right]+\varepsilon , & r\leq 2^{1/6}\sigma \\ 0, & r>2^{1/6}\sigma \end{array}\right., \end{array}$$

where *r* is the center-to-center distance between a pair of monomers, and ε sets the energy scale for the repulsion.

Neighboring beads along a chain are bonded through the finitely extensible nonlinear elastic (FENE) potential [47]

(5) $$\begin{array}{c} U_{\mathrm{FENE}}\left(r\right)=\left\{\begin{array}{cc} -\frac{kr_{0}^{2}}{2}\ln\left[1-{\left(\frac{r}{r_{0}}\right)}^{2}\right], & r<r_{0}\\ \infty , & r\geq r_{0} \end{array}\right., \end{array}$$

with spring constant $k=30\;\varepsilon /\sigma^{2}$ and maximum bond extension $r_{0}=1.5\;\sigma$ to prevent unphysical chain crossing. All parameters in Equations (4) and (5) are chosen the same for both chain types, resulting in a bond length $\ell_{b}\approx 0.97\pm 0.03\;\sigma$.

**Figure 1:**
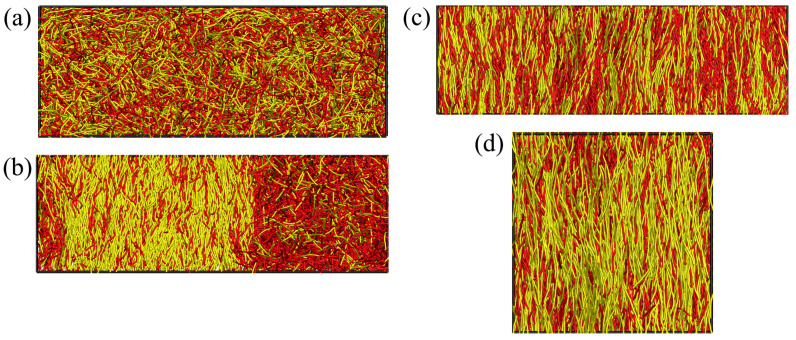
(**a**–**c**) Snapshot pictures of chain configurations from NPT runs for N=32, $\kappa_{A}=24$, and $\kappa_{B}=128$ in an elongated box geometry $L_{x}=3L_{y}=3L_{z}$, for pressures (**a**) $P=0.04\;k_{B}T/\sigma^{3}$, (**b**) $0.10\;k_{B}T/\sigma^{3}$, and (**c**) $0.27\;k_{B}T/\sigma^{3}$. Case (**a**) shows the mixed isotropic region, and case (**b**) is a state of i–n${}_{2}$ two-phase coexistence, while case (**c**) shows a mixed nematic state. The stiffer B component is shown in yellow. (**d**) Snapshot picture for $N_{A}=16$, $N_{B}=64$, and $\kappa_{A}=\kappa_{B}=128$ at $P=0.10\;k_{B}T/\sigma^{3}$. The longer B component is shown in yellow. All snapshots are for the mole fraction $X_{B}=0.5$.

MD simulations were performed either in the NVT ensemble (N being the total number of monomeric units) with a Langevin thermostat [47], or in the NPT ensemble [48,49] using the HOOMD-blue software package (v. 2.9.4) [50]. A time step of $\Delta t=0.005\;\tau_{\mathrm{MD}}$ was chosen to numerically integrate the equations of motion, with intrinsic MD time unit $\tau_{\mathrm{MD}}=\sqrt{m\sigma^{2}/\varepsilon }$. To achieve thermal equilibrium and reach the desired statistical accuracy, $10^{8}$ to $10^{9}$ integration steps were performed. Initial configurations were created by placing the A and B chains in rod-like configurations, perfectly stretched out in the *z*-direction (all bond angles being zero). The systems contained typically $10^{5}$ to $10^{6}$ monomeric units, depending on the desired average density ρ and composition $X_{B}$. Periodic boundary conditions were employed in all Cartesian directions, and the size of the simulation box was chosen distinctly larger than the polymer contour lengths *L* to avoid significant finite size effects. Figure 1 shows examples of snapshot pictures of the simulated systems.

In the MD simulations, mean square end-to-end distances $\langle R^{2}\rangle$ and gyration radii $\langle R_{g}^{2}\rangle$ are also obtained for both chain types. In nematic phases, components parallel (${\langle R^{2}\rangle }_{\|}$, ${\langle R_{g}^{2}\rangle }_{\|}$) and perpendicular (${\langle R^{2}\rangle }_{⊥}$, ${\langle R_{g}^{2}\rangle }_{⊥}$) to the director must then be distinguished. We chose the coordinate system such that the nematic director lies parallel to the *z*-axis, so that ${\langle R^{2}\rangle }_{\|}\equiv {\langle R^{2}\rangle }_{z}$ and ${\langle R^{2}\rangle }_{⊥}\equiv \left({\langle R^{2}\rangle }_{x}+{\langle R^{2}\rangle }_{y}\right)/2$ (and analogous for $\langle R_{g}^{2}\rangle$).

We also consider the single-chain structure factor [15,45]

(6) $$G\left(q\right)=\frac{1}{N}\langle \sum_{j=1}^{N}\sum_{k=1}^{N}\exp\left[iq\cdot \left(r_{j}-r_{k}\right)\right]\rangle .$$

For small $\left|q\right|$, we have the expansions [51]

(7) $$G_{\|}\left(q_{\|}\right)=N\left(1-q_{\|}^{2}{\langle R_{g}^{2}\rangle }_{\|}\right),$$

(8) $$G_{⊥}\left(q_{⊥}\right)=N\left(1-q_{⊥}^{2}{\langle R_{g}^{2}\rangle }_{⊥}\right).$$

Again, indices A and B need to be introduced in the mixture for the two types of polymers appropriately. Finally, we mention that both the tangent hard sphere chain and the bead spring model with bending potential $U_{\mathrm{bend}}$ (see Equation (1)), can be considered as discretized versions of the Kratky-Porod worm-like chain model [52,53]. The latter model is recovered in the limit N→∞, $\ell_{b}\to 0$, and σ→0, while keeping the contour length $L=\left(N-1\right)\ell_{b}$ fixed.

### 2.2. DFT Methods

For semiflexible polymers, DFT expresses the free energy of the system as a functional of the orientational distribution function f(ω). Here, ω is a shorthand for the two polar angles ϑ and ϕ describing the orientation of a molecule, which is defined by the unit vector belonging to the smallest eigenvalue of the moment of inertia tensor [54]. The theory extends Onsager’s theory for lyotropic solutions of hard rods [22] and describes the nonuniform molecular density as a product of the average density $\rho_{\mathrm{mol}}=N_{p}/V$ and f(ω), with number of polymers in the system $N_{p}$ and system volume *V*. The Helmholtz free energy per molecule is then split into an ideal contribution

(9) $$F_{\mathrm{id}}/\left(N_{p}k_{B}T\right)=\ln\left(\rho_{\mathrm{mol}}\right)-1+\int d\omega f\left(\omega \right)\ln\left[4\pi f\left(\omega \right)\right],$$

and an excess contribution

(10) $$F_{\mathrm{exc}}/\left(N_{p}k_{B}T\right)=\frac{1}{2}\rho_{\mathrm{mol}}\int d\omega \int d\omega^{\prime }f\left(\omega \right)f\left(\omega^{\prime }\right)V_{\mathrm{excl}}\left(\omega ,\omega^{\prime }\right),$$

where $V_{\mathrm{excl}}\left(\omega ,\omega^{\prime }\right)$ is the effective excluded volume between two semiflexible polymers. One can simplify the description further noting that $V_{\mathrm{excl}}$ depends only on the relative angle γ between the two chains. While $V_{\mathrm{excl}}\left(\gamma \right)$ can be computed analytically for two isolated hard rods [22], the conformational degrees of freedom of semiflexible polymers necessitate a numerical approach. Following Fynewever and Yethiraj [54], we performed additional two-chain Monte Carlo (MC) simulations to determine $V_{\mathrm{excl}}\left(\gamma \right)$ for the AA, AB, and BB chain pairs (see Refs. [37,54] for more details). Note that computing $V_{\mathrm{excl}}\left(\gamma \right)$ from two interacting chains at infinite dilution and using it in the approximation based on the second virial coefficient, Equation (10), is self-consistent only in dilute solutions. To extend the approach to larger densities $\rho_{\mathrm{mol}}$, the prefactor of $V_{\mathrm{excl}}\left(\gamma \right)$ is enhanced by Parsons-Lee [55,56] rescaling. Clearly, this procedure is purely heuristic, but extensive comparisons with MD simulations have shown that the accuracy of these approximations is reasonable at least for single-component solutions [9,38,39]. Finally, note that the nematic order parameter $S^{p}$ derived in this coarse-grained representation refers to the chain ordering as a whole, and not to the order parameter *S* associated with individual bond vectors.

### 2.3. Nematic-Nematic Phase Separation in Binary Mixtures of Semiflexible Polymers Described by the Random Phase Approximation

In our preliminary communication [36], we have presented evidence that a homogeneously mixed binary nematic phase can phase separate into A- and B-rich nematic phases at a critical point (see Figure 1). Thus, one expects that associated critical fluctuations should occur in the homogeneous phase near this critical point. In this section, we present a phenomenological (mean-field type) treatment of these critical phenomena within the random phase approximation (RPA) [12,15,57,58], to guide the analysis of the DFT and MD numerical work.

The RPA considers the collective structure factor $G_{\mathrm{coll}}\left(q\right)$, which experimentally is accessible by scattering under wavevector q. In the case of an incompressible mixture, RPA relates $G_{\mathrm{coll}}\left(q\right)$ to the single-chain structure factors $G_{A}\left(q\right)$ and $G_{B}\left(q\right)$ as

(11) $$G_{\mathrm{coll}}{\left(q\right)}^{-1}={\left[\phi_{A}G_{A}\left(q\right)\right]}^{-1}+{\left[\phi_{B}G_{B}\left(q\right)\right]}^{-1}-2\chi_{\mathrm{eff}}.$$

Here, $\chi_{\mathrm{eff}}$ is the appropriate effective FH interaction parameter, and $\phi_{A}$ and $\phi_{B}$ are the volume fractions of A and B chains, respectively, with $\phi_{A}+\phi_{B}=1$. In a symmetric mixture $\left(N_{A}=N_{B}=N\right)$ that is nematically ordered, Equations (6)–(8) and (11) yield

(12) $$G_{\mathrm{coll}}^{-1}\left(q\right)=G_{\mathrm{coll}}^{-1}\left(0\right)\left(1+\xi_{\|}^{2}q_{\|}^{2}+\xi_{⊥}^{2}q_{⊥}^{2}\right),$$

where the quantity $G_{\mathrm{coll}}^{-1}\left(0\right)$ describes the volume fraction fluctuations,

(13) $$G_{\mathrm{coll}}^{-1}\left(0\right)={\left(N\phi_{A}\right)}^{-1}+{\left(N\phi_{B}\right)}^{-1}-2\chi_{\mathrm{eff}}.$$

The correlation lengths $\xi_{\|}$, $\xi_{⊥}$ of these volume fraction fluctuations parallel and perpendicular to the director of the nematic ordering field are

(14) $$\xi_{\|}^{2}=\frac{G_{\mathrm{coll}}\left(0\right)}{N}\left(\frac{{\langle R_{g}^{2}\rangle }_{\|}^{A}}{\phi_{A}}+\frac{{\langle R_{g}^{2}\rangle }_{\|}^{B}}{\phi_{B}}\right),$$

(15) $$\xi_{⊥}^{2}=\frac{G_{\mathrm{coll}}\left(0\right)}{N}\left(\frac{{\langle R_{g}^{2}\rangle }_{⊥}^{A}}{\phi_{A}}+\frac{{\langle R_{g}^{2}\rangle }_{⊥}^{B}}{\phi_{B}}\right).$$

Equation (13) implies that the critical composition is $\phi_{A}=\phi_{B}=1/2$ and criticality occurs for $\chi_{\mathrm{eff}}=\chi_{c}=2/N$ [11,12,15]. Therefore, one finds

(16) $$\xi_{\|}^{2}={\hat{\xi }}_{\|}^{2}{\left(1-\chi_{\mathrm{eff}}/\chi_{c}\right)}^{-1},\;\xi_{⊥}^{2}={\hat{\xi }}_{⊥}^{2}{\left(1-\chi_{\mathrm{eff}}/\chi_{c}\right)}^{-1},$$

the critical amplitudes ${\hat{\xi }}_{\|}$, ${\hat{\xi }}_{⊥}$ then being related to the averages of the corresponding mean square gyration radii components

(17) $${\hat{\xi }}_{\|}^{2}=\left({\langle R_{g}^{2}\rangle }_{\|}^{A}+{\langle R_{g}^{2}\rangle }_{\|}^{B}\right)/2,\;{\hat{\xi }}_{⊥}^{2}=\left({\langle R_{g}^{2}\rangle }_{⊥}^{A}+{\langle R_{g}^{2}\rangle }_{⊥}^{B}\right)/2.$$

For a well-ordered nematic system, a chain occupies a region that can be approximately described as a cylinder of height $H=N\ell_{b}$ and radius $R=1/{\left(\pi \ell_{b}\rho \right)}^{1/2}$. This estimate comes from the condition that the volume of the cylinder $\pi R^{2}H$ contains only the *N* monomeric units of the chain, so the density inside of the cylinder is just the average density [38,39]. Hence, for dense systems, $\xi_{\|}$ is of order *L*, while $\xi_{⊥}$ is of order $\ell_{b}$. However, for very stiff chains with $\ell_{p}\gg L\gg \ell_{b}$, we know that the i–n transition occurs already for densities ρ of order 1/N, and then also the density, where n${}_{1}$–n${}_{2}$ unmixing sets in, is of the same order. Then, we predict ${\hat{\xi }}_{⊥}\propto R\propto \ell_{b}\sqrt{N}$, while ${\hat{\xi }}_{\|}$ always is of order $\ell_{b}N$. Thus, RPA predicts critical fluctuations of a very anisotropic character, and this consideration motivates some of the analyses that will be discussed in the next section.

## 3. DFT Predictions of Possible Phase Diagrams

**Figure 2:**
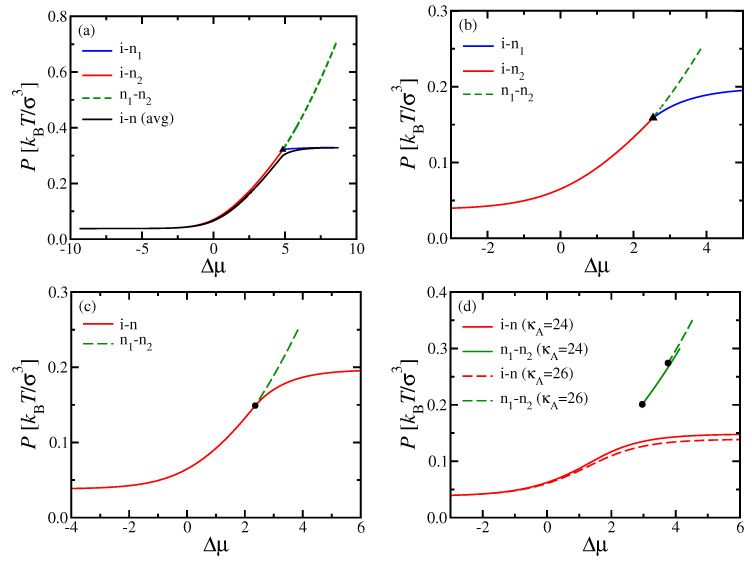
Phase diagrams of binary mixtures of two semiflexible polymers A, B with the same chain length $N_{A}=N_{B}=32$ but different stiffnesses κ. Results plotted in the variables pressure *P* versus chemical potential difference Δμ, as predicted by DFT. Results shown for (**a**) $\kappa_{A}=16$, (**b**) 20, (**c**) 20.5, and (**d**) 24 and 26 at fixed $\kappa_{B}=128$ throughout. Triple points in (**a**,**b**) are indicated by triangles, while critical points in (**c**,**d**) are denoted by dots.

Figure 2 shows phase diagrams in the space of the intensive thermodynamic variables pressure, *P*, and chemical potential difference, Δμ, for $N_{A}=N_{B}=N=32$. When we fix $\kappa_{B}$ and vary $\kappa_{A}$, there is a particular “multicritical” value $\kappa_{A}^{M}$ (estimated here as $\kappa_{A}^{M}=20.5$ for $\kappa_{B}=128$ and N=32), where the topology of the phase diagram changes: For $\kappa_{A}<\kappa_{A}^{M}$, the (first-order) transition between two distinct nematic phases ends at a triple point, where (first-order) i–n phase separations into A- or B-rich nematic phases set in. However, for $\kappa_{A}>\kappa_{A}^{M}$, the first-order n${}_{1}$–n${}_{2}$ phase separation ends at a critical point instead.

It is interesting that, for $\kappa_{A}<\kappa_{A}^{M}$, the two first-order lines for the i–n transitions meet in the (P,Δμ) plane at the triple point under some angle, while, for $\kappa_{A}=\kappa_{A}^{M}$, all transition lines seem to have a common tangent at the triple point. Note also that the value $\kappa_{A}^{M}$ where the phase diagram topology changes is not universal but depends on both $\kappa_{B}$ and *N* [36]; qualitatively similar results were found in related work for shorter chains (N=16) [37], but there the critical point occurred at distinctly larger pressures, where the unmixing of nematic phases could be potentially preempted by the appearance of smectic and/or crystalline phases. The polymers with N=32 studied here are just large enough so that one can expect n${}_{1}$–n${}_{2}$ critical points at monomer densities that are low enough so that simulation studies, and perhaps also experiments, become feasible.

**Figure 3:**
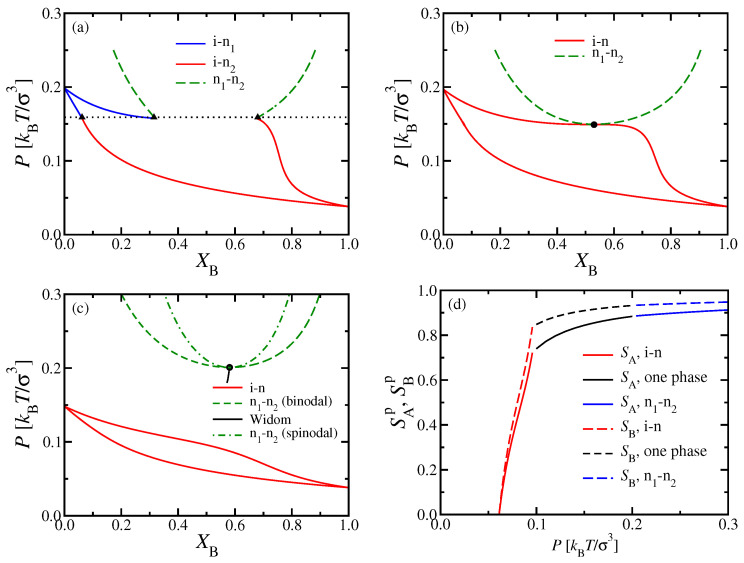
Same as Figure 2, but in the variables pressure *P* versus mole fraction $X_{B}$. Results shown for (**a**) $\kappa_{A}=20$, (**b**) 20.5, and (**c**) 24, with $\kappa_{B}=128$ and $N_{A}=N_{B}=32$ fixed throughout. The three triangles marking the location of the triple point in (**a**) have the coordinates $P_{t}=0.159\;k_{B}T\sigma^{-3}$ (dotted horizontal line) and $X_{B,t}^{i}=0.062$, $X_{B,t}^{n_{1}}=0.315$, and $X_{B,t}^{n_{2}}=0.68$. In (**c**), both the n${}_{1}$–n${}_{2}$ coexistence curve and associated mean-field spinodal are included, as well as the Widom line. Panel (**d**) shows molecular nematic order parameters $S_{A}^{p},S_{B}^{p}$ versus *P* for $X_{B}=0.5$ and $\kappa_{A}=24$.

These special features have their counterparts in the phase diagrams, where one chooses $X_{B}$ rather than Δμ as a variable (Figure 3). At the multicritical point $\kappa_{A}=\kappa_{A}^{M}$, the phase boundary of the i–n coexistence region has a horizontal tangent on the nematic side and touches the n${}_{1}$–n${}_{2}$ two-phase region at its critical point. For $\kappa_{A}>\kappa_{A}^{M}$, however, there is a single lens-shaped i–n coexistence region, which extends from the pure A system ($X_{B}=0$) to the pure B system ($X_{B}=1$). In this representation, the n${}_{1}$–n${}_{2}$ coexistence curve has an approximate parabolic shape near the critical point, which occurs at the minimum of this curve in the $\left(P,X_{B}\right)$ plane. Such a parabolic shape reflects a mean-field critical exponent, as expected for DFT. For $\kappa_{A}>\kappa_{A}^{M}$, this coexistence region no longer interferes with i–n coexistence. With increasing *P*, the n${}_{1}$–n${}_{2}$ coexistence will end when other phases (smectic liquid crystalline or crystalline solid phases) come into play, but such phases can not be captured by the present DFT treatment.

In mean-field theories of critical unmixing of binary mixtures, the response function [59]

(18) $$C={\left(\partial X_{B}/\partial \Delta \mu \right)}_{P,T}$$

not only diverges at the critical point $\left(X_{B}^{c},P_{c}\right)$, but also along the whole “spinodal curve” $X_{B}^{\mathrm{sp}}\left(P\right)$, which touches the coexistence curve at the critical point. In the phenomenological FH theory discussed in Section 2.3, where χ rather than *P* was used as a control parameter, the spinodal curve is simply given when we require $G_{\mathrm{coll}}^{-1}\left(0\right)=0$ in Equation (13), since $C\propto G_{\mathrm{coll}}\left(0\right)$ [59,60]. Although the concept of a spinodal curve is doubtful outside of mean-field theories [61], we have included its location in Figure 3c nevertheless. Generally of interest, however, is the so-called “Widom line”, describing the locus of maxima of $C(X_{B},P)$; a short piece of this line is also shown in Figure 3c. Figure 3d shows the molecular nematic order parameters $S_{A}^{p}$ and $S_{B}^{p}$ of the two types of chains plotted versus the pressure *P*, for a mole fraction $X_{B}=0.5$.

**Figure 4:**
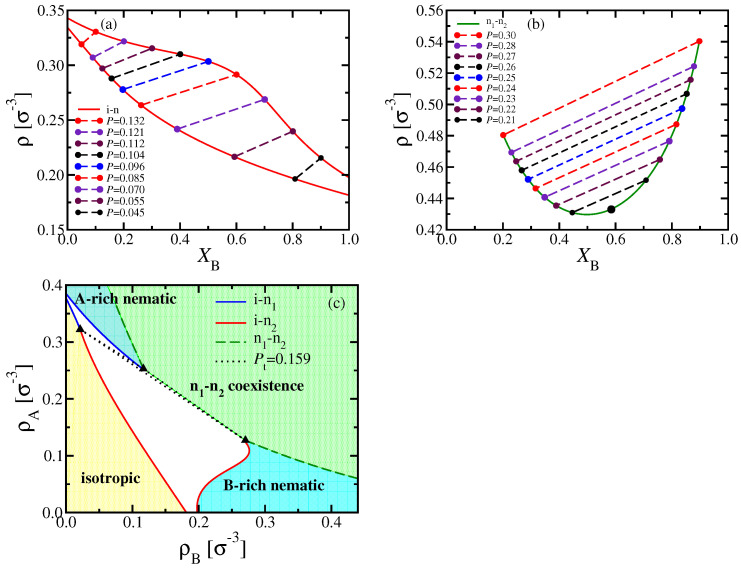
Phase diagrams of binary mixtures of two semiflexible polymers with (**a**,**b**) $\kappa_{A}=24$, and (**c**) $\kappa_{A}=20$ at fixed $N_{A}=N_{B}=32$ and $\kappa_{B}=128$. Results shown in the variables (**a**,**b**) ρ versus $X_{B}$, and (**c**) $\rho_{A}$ versus $\rho_{B}$ (cf. Figure 2d, Figure 3c and Figure 2b, Figure 3a). In (**a**,**b**) coexisting phases are highlighted as dots on the coexistence curves, while tie lines between them are indicated as dashed straight lines, at different values of the pressure *P*, as indicated. The large circle in (**b**) indicates the location of the critical point. Part (**c**) shows that the triple point of Figure 2b and corresponding triple line of Figure 3a in this representation correspond to a three-phase triangle, enclosed by the three dotted straight lines, with triangles marking its corners. The region at small densities near the origin up to the full curves contains the isotropic phase; for large $\rho_{B}$ and small $\rho_{A}$, there is a homogeneous nematic phase rich in B chains; for large $\rho_{A}$ and small $\rho_{B}$, there is a homogeneous nematic phase rich in A chains. In between the two dashed green curves, n${}_{1}$–n${}_{2}$ two phase coexistence occurs. The white region underneath the three-phase triangle is the isotropic-B-rich nematic two-phase region, and above it the isotropic-A-rich nematic two-phase region.

A clear advantage of DFT is that it is straightforward to discuss the phase behavior of the considered systems in various ensembles of statistical mechanics, which is particularly useful for making contact with experiments: The (osmotic) pressure *P* of a polymer solution is usually not readily accessible, and one rather uses the polymer concentrations as variables. In our implicit solvent model, these concentrations translate into the monomer number densities $\rho_{A}$ and $\rho_{B}$, respectively. Alternatively, we may take the total density $\rho =\rho_{A}+\rho_{B}$ and the mole fraction $X_{B}=\rho_{B}/\rho$ as variables to draw the phase diagram (Figure 4a,b).

While coexisting phases in equilibrium must be at the same temperature and pressure, their densities can differ, as clearly shown in Figure 4a,b. Note also that the critical point no longer coincides with the minimum of the two-phase coexistence curve of n${}_{1}$–n${}_{2}$ phase separation in the $\left(X_{B},\rho \right)$ phase diagram. While the tie lines, connecting the coexisting phases, are almost parallel to each other for n${}_{1}$–n${}_{2}$ coexistence, this is not true for i–n coexistence: In this case, the tie lines must get perpendicular to the $X_{B}$-axis when $X_{B}\to 0$ and $X_{B}\to 1$, but are much flatter in between. To avoid a too confusing picture, we have not shown any tie lines in Figure 4c, where the phase diagram is shown in the $\left(\rho_{A},\rho_{B}\right)$ plane; in that representation, three phases must coexist for any state within the triangle formed by the three dotted lines enclosing the triple region.

**Figure 5:**
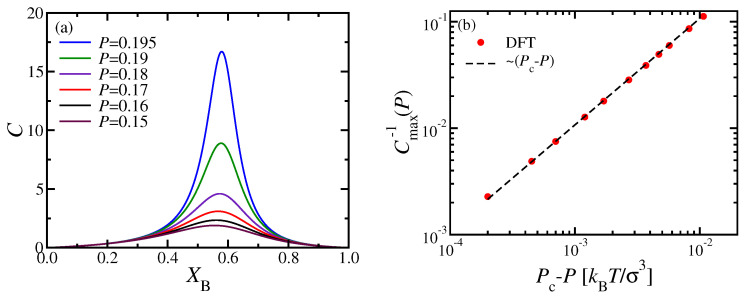
(**a**) Response function $C(X_{B},P)$ (Equation (18)) versus $X_{B}$ for the case $N_{A}=N_{B}=32$, $\kappa_{A}=24$, $\kappa_{B}=128$ for several pressures *P*, as indicated. (**b**) Log-log plot of $C_{\max}^{-1}\left(P\right)$ versus $P_{c}-P$, for the data shown in panel (**a**), using $P_{c}=0.2\;k_{B}T\sigma^{-3}$. The dashed line indicates the critical exponent γ=1 in the power law $C_{\max}\propto {\left(P_{c}-P\right)}^{-\gamma }$.

In Figure 5a, we present the response function *C* (Equation (18)) in the homogeneously mixed nematic phase, plotted versus $X_{B}$ for various pressures smaller than the critical pressure $P_{c}$. The coordinates of the maxima of these curves yield the location of the “Widom line” in the $\left(X_{B},P\right)$ plane, as shown in Figure 3c. The log-log plot of the inverse height of this maximum, $C_{\max}^{-1}$, reveals the expected critical variation, $C_{\max}^{-1}\propto P_{c}-P$ (Figure 5b).

## 4. Selected MD Results for the Phase Behavior of Mixtures of Semiflexible Polymers

**Figure 6:**
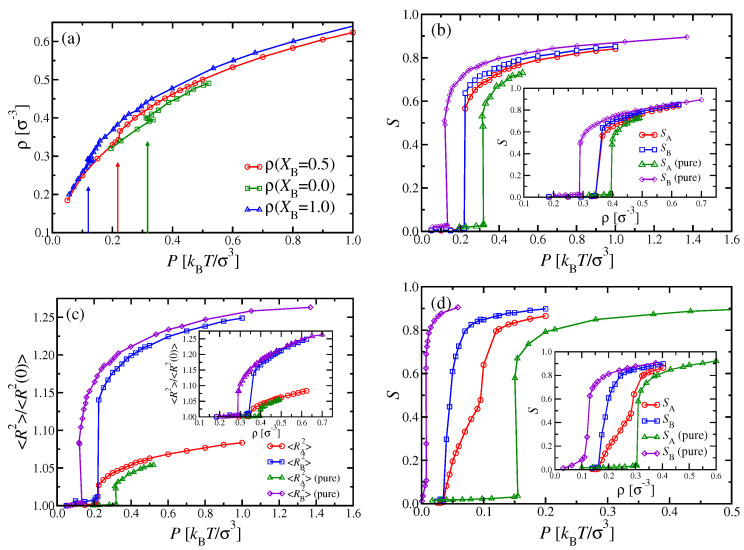
(**a**) Monomer number density ρ versus pressure *P* for the case $N_{A}=16$, $N_{B}=32$, $\kappa_{A}=\kappa_{B}=32$, for a mixture at mole fraction $X_{B}=0.5$, as well as for pure A ($X_{B}=0$) and pure B ($X_{B}=1$) solutions. (**b**) Nematic order parameters $S_{A}$ and $S_{B}$ for the systems shown in (**a**). The inset shows the same data plotted versus ρ. (**c**) Mean square end-to-end distance $\langle R^{2}\rangle$ for A and B chains in the $X_{B}=0.5$ mixture, normalized by their corresponding dilute limit $\langle R^{2}\left(\rho \to 0\right)\rangle$ versus *P*, for both A and B chains. Corresponding results for pure A and pure B solutions are also included. The inset shows the same data plotted versus ρ. (**d**) Same as (**b**), but for the case $N_{A}=16$, $N_{B}=64$, and $\kappa_{A}=\kappa_{B}=128$ at $X_{B}=0.5$.

We start this section with a rather simple special case, i.e., two types of chains with the same stiffness, $\kappa_{A}=\kappa_{B}=32$, but different chain lengths, $N_{A}=16$ and $N_{B}=32$. We have studied this system in cubic boxes, containing 12544 A and 6272 B chains ($X_{B}=0.5$). We systematically varied the pressure *P* and measured the average number density of monomeric units. Figure 6a shows the resulting equation of state, compared with the corresponding isotherms of the pure systems ($X_{B}=0$ and $X_{B}=1$). In the mixed systems, a sudden increase of the density from $\rho \approx 0.342\;\sigma^{-3}$ to $\rho \approx 0.366\;\sigma^{-3}$ takes place at $P\approx 0.215-0.220\;k_{B}T\sigma^{-3}$, where the nematic order parameters $S_{A}$ and $S_{B}$ (Figure 6b) and end-to-end distances (Figure 6c) indicate the transition, as well.

While pure systems in the NPT ensemble should exhibit a sharp first-order i–n transition, where the density ρ and the nematic order parameter *S* have a well-defined jump, this is not the case for mixtures: There, the phase diagram must have in general the shape of a lens, similar to the i–n two-phase coexistence region of Figure 3c. We would have a unique transition pressure $P_{\mathrm{trans}}$ only for a truly intensive thermodynamic variable, such as Δμ, as in Figure 2; however, since $X_{B}$ is formed from densities $\rho_{A}$ and $\rho_{B}$ of extensive variables, we rather have a two-phase coexistence region again for fixed $X_{B}$, with $P_{i}\left(X_{B}\right)<P<P_{n}\left(X_{B}\right)$. The fact that we seem to observe a unique, well-defined transition pressure in Figure 6 instead needs to be interpreted by the hypothesis that $P_{i}\left(X_{B}\right)$ and $P_{n}\left(X_{B}\right)$ are so close together that we cannot resolve the difference.

DFT respects these general rules from thermodynamics, but it suffers from other problems: The formulation which we used in this work does not give any information on chain linear dimensions, since it is just based on the distribution f(ω) of coarse-grained chains (see Section 2.2). As a consequence, also the nematic order parameter $S^{p}$ computed from DFT (Figure 3d) refers to the average orientation of the whole chain, and not to the orientation of individual bonds, as defined in Equation (3) and shown in Figure 6. It has been demonstrated [62] that there is a significant difference between the nematic order parameter defined from bond orientations and its counterpart from the orientation of whole chains. Thus, we do not have any DFT counterparts to the simulation data of Figure 6b,c; with respect to Figure 6a, even away from the i–n transition, the agreement is only qualitative but not quantitative.

An interesting question concerns the comparison of data for the nematic order parameters and chain radii in the nematic phase for the mixed system $\left(X_{B}=0.5\right)$ with their pure counterparts: We find that $S_{B}\left(X_{B}=0.5\right)<S_{B}\left(X_{B}=1\right)$, i.e., the admixture of the shorter chains, which exhibit somewhat less nematic order, weakens the order of the longer chains slightly. This is also evident from the fact that $S_{A}\left(X_{B}=0.5\right)<S_{B}\left(X_{B}=0.5\right)$ throughout the nematic phase. On the other hand, we also have $S_{A}\left(X_{B}=0.5\right)>S_{A}\left(X_{B}=0\right)$ for those pressures where nematic order occurs. Those chains which order better (here, the longer chains) act like a nematic ordering field on the chains which have less tendency to order (in the pure phase). We shall see later that the same phenomenon occurs when we examine mixtures of chains that have identical *N* but differ in stiffness.

Throughout the isotropic phase, we find here $\langle R_{g,A}^{2}\rangle =18.15\;\sigma^{2}$ and $\langle R_{g,B}^{2}\rangle =66.4\;\sigma^{2}$, irrespective of *P* and $X_{B}$, and likewise for the mean square end-to-end distances, $\langle R_{A}^{2}\rangle \approx 182\;\sigma^{2}$ and $\langle R_{B}^{2}\rangle \approx 669\;\sigma^{2}$. Since the persistence length is about twice as large as the contour length for the A chains, only a rather modest increase of $\langle R_{A}^{2}\rangle$ can be observed at the i–n transition (Figure 6c). In contrast, the longer B chains exhibit a marked jump of their mean square end-to-end distances at the i–n transition. Further note that $\sqrt{\langle R_{B}^{2}\rangle }$ for $P=1\;k_{B}T\sigma^{-3}$ has already reached 96% of the contour length, at which this quantity must saturate for large enough pressures.

**Figure 7:**
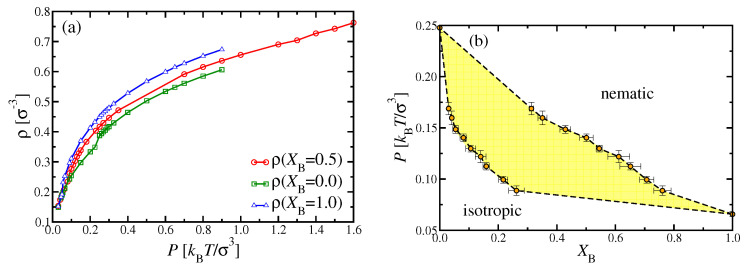
(**a**) Monomer number density ρ versus pressure *P* for systems with $N_{A}=N_{B}=32$, and $\kappa_{A}=24$, $\kappa_{B}=128$. Data recorded in the NPT ensemble at three compositions, $X_{B}=0$, $X_{B}=0.5$, and $X_{B}=1$. (**b**) Phase diagram of the same system as in (**a**), in the plane of variables *P* and $X_{B}$. Lines are guides to the eye only. Error bars of the pressure refer to the fluctuations of the pressure measured via the virial theorem.

Now, we turn to some of the cases that were already studied by DFT in Section 3, where chains have the same chain length $N_{A}=N_{B}=32$, but differ in stiffness, e.g., $\kappa_{A}=24$ and $\kappa_{B}=128$. From DFT, we would expect a transition from the isotropic phase into the i–n two-phase coexistence region with increasing pressure, then a nematic phase to which both types of chains contribute, followed by n${}_{1}$–n${}_{2}$ unmixing at still higher pressures (Figure 3c). Figure 7a shows the density versus pressure curve of the equimolar mixture ($X_{B}=0.5$) from MD simulations, compared to the corresponding pure systems. For the latter, the i–n transitions show up as little kinks at $P_{\mathrm{trans}}\approx 0.24\;k_{B}T\sigma^{-3}$ and $0.07\;k_{B}T\sigma^{-3}$ for the A and B chains, respectively, with corresponding monomer number densities being $\rho_{i}\approx 0.37\;\sigma^{-3}$ and $\rho_{n}\approx 0.38\sigma^{-3}$ for species A, while $\rho_{i}\approx \rho_{n}\approx 0.21\;\sigma^{-3}$ for species B. Unlike DFT, we cannot resolve the density jump at the transition with meaningful accuracy in the MD simulations. Nevertheless, the corresponding DFT results for the transition pressures, i.e., $P_{\mathrm{trans}}=0.145\;k_{B}T\sigma^{-3}$ and $0.035\;k_{B}T\sigma^{-3}$ for the pure A and B systems, respectively, are clearly far outside of the MD error bars. Thus, there is almost a discrepancy by a factor of two in the pressure scale. However, the underestimation by DFT with respect to the densities is much smaller (Figure 4). When we examine the ρ versus *P* curve for the mixture, the i–n transition cannot be recognized from the data at all (Figure 7a). This behavior can be expected when the two-phase coexistence region is not very narrow (as it was in Figure 6), but rather wide, as found by DFT for the present case (Figure 3c). Then, the density variation in this two-phase region is described by the lever rule,

(19) $$\rho \left(X_{B},P\right)=\rho \left(X_{B}^{i}\left(P\right),P\right)+\frac{X_{B}-X_{B}^{i}\left(P\right)}{X_{B}^{n}\left(P\right)-X_{B}^{i}\left(P\right)}\rho \left(X_{B}^{n}\left(P\right),P\right),$$

where $X_{B}=X_{B}^{i}\left(P\right)$ and $X_{B}=X_{B}^{n}\left(P\right)$ describe the curves limiting the two-phase region at the isotropic and nematic side, respectively. At these curves, $\rho (X_{B},P)$ has at most a (small) discontinuity of slope, which clearly could not be seen in the simulation.

In order to obtain a simulation counterpart (Figure 7b) to the i–n miscibility gap predicted from DFT in Figure 3c, we had to carry out MD simulations for various choices of $X_{B}$, and analyze the compositions observed in suitably placed $\ell_{x}\times L_{y}\times L_{z}$ subsystems with $\ell_{x}\ll L_{x}=3L_{y}$; “suitably placed” means that simulation snapshots were inspected to check for states where two-phase coexistence occurs with interfaces perpendicular to the *x*-axis (see Figure 1b). The subsystems were then centered far from these interfaces, and compositions (as well as other observables) were recorded separately for the isotropic and nematic subsystems. To avoid systematic errors, we have checked that the diffusion of the domain walls in the *x*-direction was small enough during the time interval in which subsystem averages were taken. However, rather large statistical fluctuations of all observables are inevitable in such simulations of two-phase coexistence. Further note that we did not succeed to prepare reasonably stable states of two-phase coexistence for pressures $P\leq 0.085\;k_{B}T\sigma^{-3}$ and $P\geq 0.175\;k_{B}T\sigma^{-3}$, but rather metastable homogeneous nematic or isotropic states took over in those pressure regions. Hence, the connections of the observed phase boundaries in Figure 7b to the pure system phase transitions at $X_{B}=0$ and $X_{B}=1$ are tentative interpolations only.

In principle, one could improve the results by running considerably larger systems and/or longer simulation times, which would, however, require a prohibitively large investment of additional computational resources. Thus, while MD simulations yield in principle the exact statistical mechanics of the considered model system, one has to be aware of its practical limitations. Nevertheless, our MD simulations confirm the DFT prediction that a rather wide i–n miscibility gap occurs for intermediate values of $X_{B}$ (Figure 3c), unlike the system shown in Figure 6. Figure 7b suggests that, for $X_{B}=0.5$, the two-phase region extends from about $P\approx 0.08\;k_{B}T\sigma^{-3}$ to about $0.14\;k_{B}T\sigma^{-3}$, compatible with Figure 7a.

Since DFT predicted for the case $\kappa_{A}=24$, $\kappa_{B}=128$ a region of n${}_{1}$–n${}_{2}$ unmixing for $P>P_{c}\approx 0.2\;k_{B}T\sigma^{-3}$ (Figure 3c), or $\rho >\rho_{c}\approx 0.43\;\sigma^{-3}$ (Figure 4b), we extended our MD studies to significantly larger pressures (and corresponding densities) than shown in Figure 7, in order to search for MD evidence of n${}_{1}$–n${}_{2}$ phase separation in this model. To this end, we performed additional simulations in elongated boxes of size 128σ×64σ×64σ, with hard walls placed at x=−64σ and x=+64σ to stabilize nematic order. Starting configurations were generated by orienting all chains along the *z*-axis, and placing the A and B chains in the right (x>0) and left half (x<0) of the simulation box, respectively. However, we found that the initially separated A- and B-rich phases completely decay by interdiffusion. Even for $\rho =0.65\;\sigma^{-3}$, a state deep in the nematic phase for both pure A and pure B systems, the system still develops towards a homogeneous mixture in the region far from the confining walls. At still larger densities, the system is too slowly relaxing to clearly establish equilibrium, and smectic order starts to take over in the pure B phase [40].

Since the occurrence of n${}_{1}$–n${}_{2}$ unmixing can also be seen already in the homogeneous phase by a growth of the response function $C(X_{B},P)$ as $P\to P_{c}$ (Equation (18)), cf. Figure 5a, we attempted to estimate this quantity in the density regime where we still can equilibrate well with MD, namely $0.42\;\sigma^{-3}\leq \rho \leq 0.57\;\sigma^{-3}$. To mimic a grandcanonical ensemble in our MD simulations where the total numbers of A and B chains are strictly fixed, we study fluctuations of $X_{B}$, as described by $C(X_{B},P)$, in small subsystems of the total system. In any such subsystem, the volume fraction is not conserved: Because the remainder of the system acts like a reservoir on the subsystem, one essentially realizes a grandcanonical ensemble for the subsystem. The feasibility of such an approach has been demonstrated previously for both the Lennard-Jones fluid at constant density [63] and the Ising/lattice gas model [64,65].

**Figure 8:**
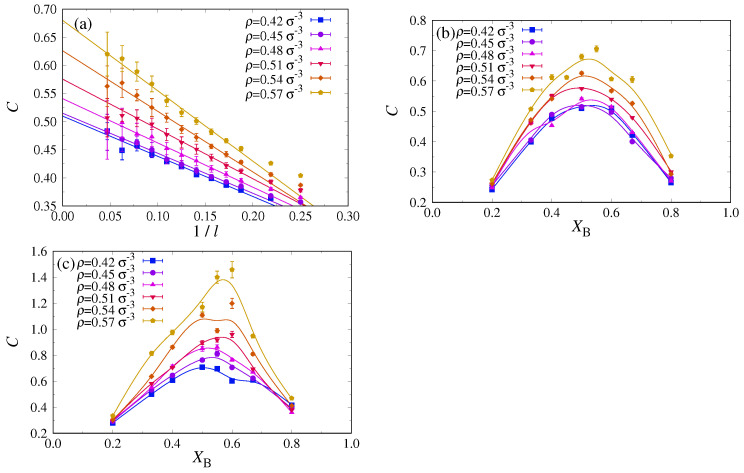
(**a**) Response function $C(X_{B},\rho )$ measured in ℓ×ℓ×σ subsystems of a system with $L_{x}=L_{y}=L_{z}=64\;\sigma$, $\kappa_{A}=24$, $\kappa_{B}=128$, $N_{A}=N_{B}=32$, at $X_{B}=0.5$, plotted versus 1/ℓ. The dotted straight lines indicate the linear extrapolation towards 1/ℓ→0. Six densities from $\rho =0.42\;\sigma^{-3}$ to $0.57\;\sigma^{-3}$ are included, as indicated. (**b**) Extrapolated response functions $C(X_{B},\rho )$ as obtained in (**a**) versus $X_{B}$ for various ρ, as indicated. Curves are intended as guides to the eye only. (**c**) Same as (**b**), but using a more flexible A component, $\kappa_{A}=20$.

For this subsystem analysis, it is most convenient to work in the NVT ensemble and choose a cubic simulation box with $L_{x}=L_{y}=L_{z}$. For an Ising system, it would be adequate to choose subsystems of the same shape, i.e., a cubic volume ℓ×ℓ×ℓ with $\ell \ll L_{z}$. However, in the present case of nematically ordered systems, we must be aware of an extremely strong anisotropy: Since the A and B chains are stretched out over a length of the order of $L\approx \left(N-1\right)\ell_{b}$ along the *z*-axis, all volume fraction fluctuations in the *z*-direction are strongly correlated; on a mean-field level, this effect was already described via the distinction of the correlation lengths $\xi_{\|}$ and $\xi_{⊥}$ (see Equations (14)–(17)). To test whether there is a tendency in favor of phase separation in the lateral directions, *x* and *y*, we found it advantageous to study quasi-two dimensional subsystems perpendicular to the *z*-axis. The resulting fluctuations $C\left(X_{B},P\right)=G_{\mathrm{coll}}\left(X_{B},0\right)$ are plotted in Figure 8a versus 1/ℓ, since one expects a (1/ℓ)-finite size correction due to the subsystem boundary [63,64,65]. The data are compatible with this expectation but also show that there is only a very modest increase of $G_{\mathrm{coll}}\left(X_{B}=0.5,0\right)$ with increasing density (Figure 8a). This is also true for other choices of $X_{B}$ (Figure 8b), confirming our conclusion that the blend $\kappa_{A}=24$, $\kappa_{B}=128$ does not undergo critical n${}_{1}$–n${}_{2}$ unmixing at densities where one still has the nematic phase.

Next, we studied systems with slightly more flexible A chains ($\kappa_{A}=20$, $\kappa_{B}=128$), which, according to our DFT calculations (cf. Figure 3a), no longer have a mixed nematic phase extending over the full range of investigated pressures $\left(0.27\;k_{B}T\sigma^{-3}<P<0.75\;k_{B}T\sigma^{-3}\right)$ and densities $\left(0.4\;\sigma^{-3}<\rho <0.6\;\sigma^{-3}\right)$. Indeed, now the fluctuation near $X_{B}=0.5$ increases distinctly stronger with increasing density (Figure 8c) than for the case $\kappa_{A}=24$. However, it turns out to be extremely difficult to reach a decent precision of these data; hence, we could still not locate the critical point of n${}_{1}$–n${}_{2}$ unmixing of the system with this method. One problem comes from the fact that critical slowing down is known to lead to a systematic bias (underestimation) of $G_{\mathrm{coll}}\left(q=0\right)$ when the runs are not long enough [66]. This problem is particularly severe for critical binary mixtures, where a finite size scaling of the relaxation time for interdiffusion $\tau_{\mathrm{int}}\propto \ell^{4}$ is predicted [67].

**Figure 9:**
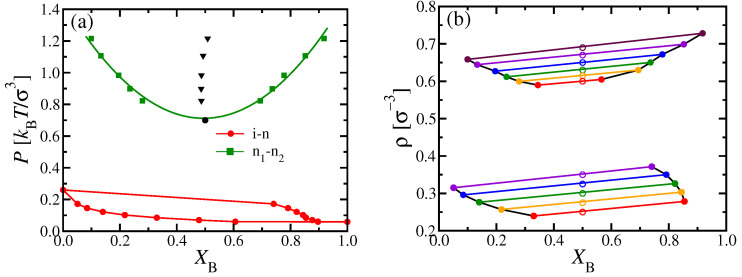
Phase diagram of the system $N_{A}=N_{B}=32$, $\kappa_{A}=20$, $\kappa_{B}=128$ shown in the variables pressure *P* versus mole fraction $X_{B}$. The location of the rectilinear diameter is indicated by black triangles, while the estimate $P_{c}\approx 0.73\;k_{B}T\sigma^{-3}$ for the location of the critical point is highlighted by a black dot. (**b**) Data showing the total densities in the coexisting phases corresponding to (**a**). The lower set of points shows data referring to i–n${}_{2}$ phase coexistence, the upper set n${}_{1}$–n${}_{2}$ phase coexistence. The coexisting phases are connected by the straight tie lines. Each tie line corresponds to a chosen average density ρ at $X_{B}=0.5$, which are highlighted by open symbols. Curves are guides to the eye only.

**Figure 10:**
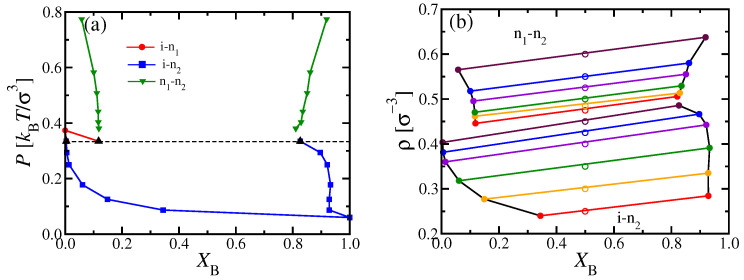
(**a**) Same as Figure 9a, but for $\kappa_{A}=16$. Here, i–n${}_{2}$ phase coexistence is shown by blue squares, n${}_{1}$–n${}_{2}$ phase coexistence by green triangles, and i–n${}_{1}$ phase coexistence by red dots. The pressure at the triple point is indicated by a horizontal dashed line and the points where phase boundaries end there by black triangles. The triple point pressure is at $P_{t}\approx 0.32\;k_{B}T\sigma^{-3}$. (**b**) Data showing the total densities in the coexisting phases corresponding to panel (**a**). The lower set of points shows data corresponding to i–n${}_{2}$ phase coexistence, the upper set n${}_{1}$–n${}_{2}$ phase coexistence. No data for i–n${}_{1}$ phase coexistence are included, and the intermediate i–n${}_{1}$–n${}_{2}$ three-phase triangle is also not shown. The coexisting phases are connected by (straight) tie lines. Each tie line corresponds to a chosen average density at $X_{B}=0.5$, which are highlighted by open symbols. Curves are guides to the eye only.

However, we found it possible to study n${}_{1}$–n${}_{2}$ unmixing for $\kappa_{A}=20$, $\kappa_{B}=128$, using the NVT ensemble and elongated boxes with $L_{y}=32\;\sigma$, $L_{z}=64\;\sigma$, N= 196,608, and a choice of $L_{x}$ such that the desired density was realized (e.g., $L_{x}=192\;\sigma$ for $\rho =0.5\;\sigma^{-3}$). The time step here was chosen as $0.0025\;\tau_{\mathrm{MD}}$, and runs of $2.5\times 10^{9}$ MD steps were carried out for compositions $X_{B}=1/3$, 1/2 and 2/3. Starting again the simulations with chains oriented along the *z*-axis and initially separated in pure A and B domains (according to the chosen $X_{B}$), equilibrium was reached for $\kappa_{A}\leq 20$ for the densities of interest. We then determined the phase diagrams of Figure 9 and Figure 10 by analyzing the resulting density profiles $\rho_{A}\left(x\right)$ and $\rho_{B}\left(x\right)$. From Figure 9, it is obvious that the topology of the phase diagram found in MD disagrees with its DFT counterpart for $\kappa_{A}=20$ (Figure 3a), but rather resembles the DFT phase diagram for $\kappa_{A}=24$ (Figure 3c). Likewise, the MD phase diagram for $\kappa_{A}=16$ (Figure 10) is qualitatively similar to the DFT phase diagram found for $\kappa_{A}=20$ (Figure 3a). Thus, the massive discrepancies between DFT and MD phase diagrams for choices of $\kappa_{A}=24$ and $\kappa_{A}=20$ should not be interpreted as a complete breakdown of the DFT method: Rather, the latter is only somewhat inaccurate with respect to the prediction of the value $\kappa_{A}^{M}$, where the phase diagram topology changes. For $\kappa_{A}<\kappa_{A}^{M}$, the phase diagram exhibits a triple point, while, for $\kappa_{A}>\kappa_{A}^{M}$, two separate coexistence regions occur, i.e., an i–n region at lower pressures and a n${}_{1}$–n${}_{2}$ coexistence region (ending in a critical point) at higher pressures. DFT predicts $\kappa_{A}^{M}=20.5$ for $\kappa_{B}=128$, while MD rather suggests $\kappa_{A}^{M}=18\pm 1$. Apart from this quantitative mismatch in $\kappa_{A}^{M}$, the general sequence of phase diagram changes with increasing $\kappa_{A}$ predicted by MD and DFT is the same. Further, these findings are also compatible with the predictions obtained by Semenov and Subbotin [21] for the limiting case of very large contour lengths. Hence, we conclude that the general features of the phase behavior predicted here are rather robust.

An important aspect of phase coexistence in the studied systems is that only the pressure *P* in the coexisting phases is identical, while the density ρ is not, as evidenced from the nonzero slopes of the tie lines drawn in Figure 9b and Figure 10b. This density inhomogeneity could also be seen very well in the density profiles ρ(x) in the direction perpendicular to the interfaces: Due to the periodic boundary conditions, there must be two interfaces, and the slope of the tie lines tells us that the density of the phase n${}_{2}$ is larger than both the density of the phase n${}_{1}$ and the isotropic phase. Due to this density variation at the i–n${}_{2}$ interfaces, there could occur some excess density (“interfacial adsorption”) associated with these interfaces, which might affect our analysis of the MD results (as a finite size effect of order $1/L_{x}$). However, a more detailed analysis of our results has shown that this effect (and other finite size effects) is negligible. Rather, we could show that our results on phase coexistence are fully compatible with consequences of the Gibbs phase rule. In fact, when the two A-rich (a) and B-rich (b) phases coexist in our simulation volume, the volume consists of two parts, $V=V_{a}+V_{b}$, since, per definition, interfaces have zero volume. The particle numbers $N_{A}$ and $N_{B}$ of the two kinds of monomers can be likewise split into particle numbers in the two phases

(20) $$\begin{array}{cc} N_{A} & =N_{\mathrm{Aa}}+N_{\mathrm{Ab}}, \end{array}$$

(21) $$\begin{array}{cc} N_{B} & =N_{\mathrm{Ba}}+N_{\mathrm{Bb}}, \end{array}$$

with $X_{B}=N_{B}/\left(N_{A}+N_{B}\right)$. The phase boundaries in Figure 9 and Figure 10 were based on the four partial densities $\rho_{\mathrm{Aa}}=N_{\mathrm{Aa}}/V_{a}$, $\rho_{\mathrm{Ab}}=N_{\mathrm{Ab}}/V_{b}$, $\rho_{\mathrm{Ba}}=N_{\mathrm{Ba}}/V_{a}$, $\rho_{\mathrm{Bb}}=N_{\mathrm{Bb}}/V_{b}$, namely $\rho_{a}=\rho_{\mathrm{Aa}}+\rho_{\mathrm{Ba}}$, $\rho_{b}=\rho_{\mathrm{Ab}}+\rho_{\mathrm{Bb}}$, and $X_{\mathrm{Ba}}=\rho_{\mathrm{Ba}}/\rho_{a}=N_{\mathrm{Ba}}/N_{a}$, as well as $X_{\mathrm{Bb}}=\rho_{\mathrm{Bb}}/\rho_{b}=N_{\mathrm{Bb}}/N_{b}$, with $N_{a}=N_{\mathrm{Aa}}+N_{\mathrm{Ba}}$, $N_{b}=N_{\mathrm{Ab}}+N_{\mathrm{Bb}}$. Note that the volume fraction $V_{b}/V$ must not be confused with the mole fraction $X_{B}=N_{B}/N$.

Due to Equations (20) and (21) and the relation for *V*, the four partial densities are not all independent of each other. We have found it convenient to formulate this dependence via two equations for the volume fraction of phase b, $V_{b}/V=r_{1}=r_{2}$, with ratios $r_{1}$ and $r_{2}$ defined as

(22) $$r_{1}=\left(\rho_{B}-\rho_{\mathrm{Ba}}\right)/\left(\rho_{\mathrm{Bb}}-\rho_{\mathrm{Ba}}\right),\;r_{2}=\left(\rho_{A}-\rho_{\mathrm{Aa}}\right)/\left(\rho_{\mathrm{Ab}}-\rho_{\mathrm{Aa}}\right),$$

with partial densities $\rho_{B}=N_{B}/V$ and $\rho_{A}=N_{A}/V$. Table A1 and Table A2 quote the partial densities for $X_{B}=0.5$, from which Figure 9 and Figure 10 were constructed, as well as the estimates $r_{1}$ and $r_{2}$ of the corresponding volume fraction of the B-rich phase. Results for $X_{B}=1/3$ and 2/3 were compatible with these results and are therefore not shown.

**Figure 11:**
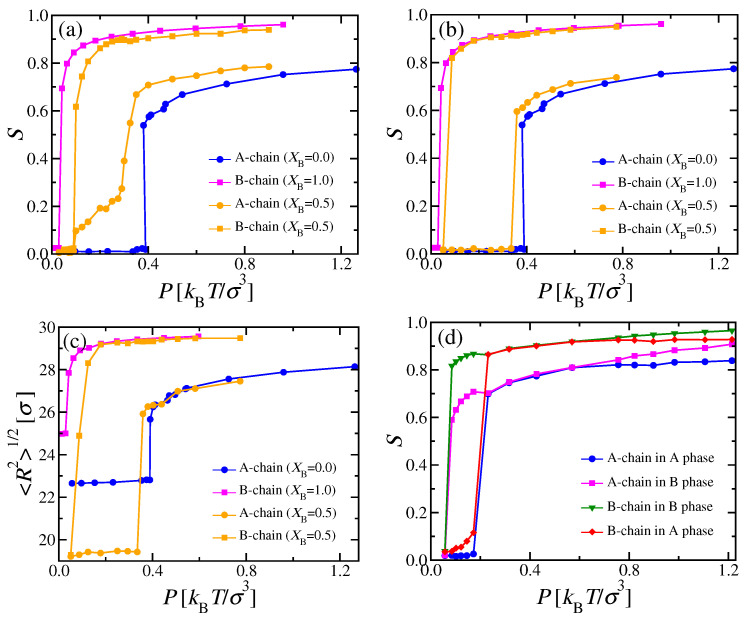
(**a**) Nematic bond order parameters $S_{A}$ and $S_{B}$ of the two types of chains A, B with $N_{A}=N_{B}=32$, $\kappa_{A}=16$ and $\kappa_{B}=128$ versus pressure *P*, for three choices of $X_{B}$, as indicated. (**b**) Same as (**a**), but $S_{A}$ and $S_{B}$ calculated only for A chains in A-rich phases, and B chains in B-rich phases. (**c**) End-to-end distances ${\langle R^{2}\rangle }^{1/2}$ versus *P*, for the same system as panel (**b**). (**d**) $S_{A}$ and $S_{B}$ versus *P* for the case $N_{A}=N_{B}=32$, $\kappa_{A}=20$, $\kappa_{B}=128$, for $X_{B}=0.5$. For $0.075\;k_{B}T\sigma^{-3}<P<0.2\;k_{B}T\sigma^{-3}$, there occurs phase separation into isotropic and nematic phases (cf. Figure 9), and, for about $0.2\;k_{B}T\sigma^{-3}<P<0.7\;k_{B}T\sigma^{-3}$, we have a uniformly mixed nematic phase, while, for about $P>0.7\;k_{B}T\sigma^{-3}$ n${}_{1}$–n${}_{2}$, unmixing has occurred.

We now discuss the nematic bond order parameters of the two types of chains, $S_{A}$ and $S_{B}$, respectively, extracted as usual [35,39] as the largest eigenvalue of the tensor $Q^{\alpha \beta }$ (Equation (3)). In the pure A and B systems, a discontinuous transition occurs at $P\approx 0.38\;k_{B}T\sigma^{-3}$ and $P\approx 0.07\;k_{B}T\sigma^{-3}$, respectively, where the nematic order parameter jumps from zero to about $S_{A}\approx 0.52$ and $S_{B}\approx 0.76$ [9,39], respectively (Figure 11a). In contrast, the mixed systems exhibit a more gradual behavior, as shown in Figure 11a. As expected from the phase diagram (Figure 10a), $S_{B}\left(X_{B}=0.5,P\right)$ starts to rise steeply as soon as the i–n${}_{2}$ two-phase coexistence region has entered around $P\approx 0.10\;k_{B}T\sigma^{-3}$. Unlike the pure B system, the increase of $S_{B}\left(X_{B}=0.5,P\right)$ is continuous, since the mole fraction of the nematic phase grows continuously from zero to one as the two-phase coexistence region is crossed. In addition, $S_{A}\left(X_{B}=0.5,P\right)$ starts to become nonzero together with $S_{B}\left(X_{B}=0.5,P\right)$, due to A chains that are dissolved in the nematic B-rich phase (at a very small mole fractions, cf. Figure 10a), and which align due to the nematic ordering field exerted by surrounding majority of B chains. At $P\approx 0.3\;k_{B}T\sigma^{-3}$, the rise of $S_{A}\left(X_{B}=0.5,P\right)$ is already rather steep: We interpret this phenomenon as a “capillary nematization” effect of the nematic B-rich domains on the remaining A-rich domains, which, in a truly macroscopic system, would still be in the isotropic phase up to a pressure of about $P\approx 0.35\;k_{B}T\sigma^{-3}$, according to the phase diagram shown in Figure 10a. This finite size effect will be discussed in more detail below.

In bulk (macroscopic) systems, the gradual increase of the order parameters can be interpreted in terms of the lever rule

(23) $$S_{A,B}\left(X_{B},P\right)=\frac{X_{B}-X_{B}^{i}\left(P\right)}{X_{B}^{n}\left(P\right)-X_{B}^{i}\left(P\right)}S_{A,B}\left(X_{B}^{n}\left(P\right)\right).$$

Here, the two boundaries of the i–n two-phase coexistence region were denoted as $X_{B}^{i}\left(P\right)$ and $X_{B}^{n}\left(P\right)$. If we could cross this region at fixed *P* by varying $X_{B}$, the variation of $S_{A}$ (or $S_{B}$, respectively) with $X_{B}$ would be simply linear. However, this clearly is not true when we vary *P* at fixed $X_{B}$ (different from $X_{B}=0$ or 1, of course): Then, $S_{A}$ and $S_{B}$ are nontrivial functions, reflecting both the variation of the phase boundaries $X_{B}^{i}\left(P\right)$ and $X_{B}^{n}\left(P\right)$, as well as of $S_{A,B}\left(X_{B}^{n}\left(P\right)\right)$.

The behavior described in Figure 11a and Equation (22) would be observed experimentally by methods that average over all domains in the system, e.g., scattering, optical birefringence, etc. In the simulations, it is possible to resolve the order parameters separately in the two kinds of domains: Then, $S_{B}\left(P\right)$ measured in the nematic B-rich domain rises much faster with *P* (Figure 11b), similar to the result of the pure B phase. Likewise, $S_{A}\left(P\right)$ measured in the isotropic A-rich domains stays zero up to $P\approx 0.35\;k_{B}T\sigma^{-3}$, where also the A-rich phase becomes nematic (Figure 11b). An analogous interpretation explains the behavior of the end-to-end distance measured separately in the coexisting domains (Figure 11c). Equation (23) applies only for pressures $P<P_{t}$ because, for larger pressures, we enter a different two-phase coexistence region between two different nematic phases. The generalization to this case is

(24) $$S_{A,B}\left(X_{B},P\right)=S_{A,B}\left(X_{B}^{n_{1}}\left(P\right)\right)+\frac{X_{B}-X_{B}^{n_{1}}\left(P\right)}{X_{B}^{n_{2}}\left(P\right)-X_{B}^{n_{1}}\left(P\right)}S_{A,B}\left(X_{B}^{n_{2}}\left(P\right)\right).$$

In the simulation, the order parameter of the less stiff chains increases indeed steeply around $P\approx 0.35\;k_{B}T\sigma^{-3}$: According to theory, there should be a clear jump of $S_{A}\left(X_{B},P\right)$ at $P_{t}$, since $S_{A}\left(X_{B}^{i}\left(P<P_{t}\right)\right)\equiv 0$ in the isotropic phase, while $S_{A}\left(X_{B}^{n_{1}}\left(P\geq P_{t}\right)\right)>0$ in the phase $n_{1}$. There is also a discontinuous increase from the composition of the isotropic phase $\left(X_{B}^{i}\left(P_{t}\right)\right)$ to the composition $X_{B}^{n_{1}}\left(P_{t}\right)$ of the A-rich nematic phase. The resulting singular behavior of $S_{A}\left(X_{B},P\right)$ and $S_{B}\left(X_{B},P\right)$ predicted by Equations (23) and (24) is somewhat smeared out in the simulations, presumably due to finite size effects. In addition, the polymer end-to-end distances (Figure 11c) do not show related singularities either. Thus, it clearly would be desirable to study the system using much larger N and much better statistics, which was, however, computationally infeasible for us. One reason for unexpectedly large finite size effects is recognized when we examine the order parameters $S_{A}^{i}$, $S_{B}^{i}$, $S_{A}^{n_{1}}$, $S_{B}^{n_{1}}$, $S_{A}^{n_{2}}$, and $S_{B}^{n_{2}}$ that belong to the various phases: We find that, in the case where we have i–n${}_{2}$ two-phase coexistence in the simulation box, the order parameter $S_{B}^{i}$ grows gradually with *P* and is of order 0.5 already when $P=P_{t}$ is reached. We interpret this finding as a “capillary nematization” effect of the B chains, which have $S_{B}^{n_{2}}\approx 0.9$ for *P* near $P_{t}$ already in the $n_{2}$ phase, in the isotropic phase adjacent to the i–n${}_{2}$ interfaces. As $P\to P_{t}$, a kind of nematic wetting layer grows in the isotropic phase at the i–n${}_{2}$ interface. If the linear dimensions $L_{x}\to \infty$, this effect will become negligibly small, but it is not for the $L_{x}$ values accessible to us. Another interesting feature is that, in the mixed nematic phase, the order parameter of the less stiff phase ($S_{A}$) always exceeds the corresponding value of the pure A phase at the same pressure, and likewise for the stiffer component ($S_{B}$) it is slightly smaller than for its pure counterpart. Thus, the more ordered stiffer chains in the direct neighborhood of less stiff chains enhance the order of the latter; conversely, the less stiff chains somewhat perturb the order of the stiffer ones, but the local nematic order of the mixture is very nonuniform since $S_{A}\left(X_{B}=0.5\right)$ is distinctly smaller than $S_{B}\left(X_{B}=0.5\right)$. A similar effect has already been noted for mixtures of chains which have the same stiffness but differ in chain length (where the longer chains are more ordered), as in Figure 6b,d.

Figure 11d shows a counterpart to Figure 11b for the case $\kappa_{A}=20$, where a uniformly mixed nematic phase occurs. While we expect a discontinuous transition from $S_{A}=0$ in the isotropic phase to a nonzero value in the uniformly mixed nematic phase at the transition from the i–n two-phase region, a continuous splitting of both $S_{A}$ and $S_{B}$ into their different values in the coexisting nematic phase is expected at $\left(X_{B}^{c},P_{c}\right)$. Indeed, the data are compatible with this prediction. We see that $S_{\mathrm{Ba}}$ in the A-rich nematic phase grows gradually from zero to distinctly nonzero values, when the transition to the nematic phase is approached: Again, we interpret this precursor effect as a finite size effect, due to “capillary nematization” at the i–n interfaces of the domain of the isotropic phase which has a finite extent in *x*-direction (according to Table A1, the volume fraction taken by the isotropic phase is about 0.39 for $\rho =0.35\;\sigma^{-3}$ ($P\approx 0.172\;k_{B}T\sigma^{-3}$)).

Finally, we make contact between the n${}_{1}$–n${}_{2}$ critical point found here (Figure 9 and Figure 11d) and the phenomenological theory of Section 2.3: Can one understand the χ parameter postulated there from the stiffness asymmetry? To answer this question, we apply the approach of Kozuch et al. [42], who had considered only isotropic mixtures of rather flexible long polymers with slightly different stiffnesses.

Noting that Equation (13) also follows from the FH free energy

(25) $$\begin{array}{c} F_{\mathrm{FH}}\left(\phi_{A},\phi_{B}\right)=k_{B}T\left[\frac{\phi_{A}}{N_{A}}\ln\phi_{A}+\frac{\phi_{B}}{N_{B}}\ln\phi_{B}+\chi_{\mathrm{eff}}\phi_{A}\phi_{B}\right], \end{array}$$

with volume fractions $\phi_{A}+\phi_{B}=1$ [11,12,15], one can see that the excess term $\chi_{\mathrm{eff}}\phi_{A}\phi_{B}$ relative to the entropy of mixing terms of the ideal mixture can only result from the difference in bending energies $U_{\mathrm{bend}}^{A}\left(\theta_{ijk}\right)$ and $U_{\mathrm{bend}}^{B}\left(\theta_{ijk}\right)$, since both types of chains are identical in all other respects for $N_{A}=N_{B}$. Since the average energy 〈E〉=∂〈βF〉/∂β (with $\beta \equiv 1/(k_{B}T)$), one can apply thermodynamic integration methods, based on the appropriate difference in bending energy. Denoting the total free energy of the mixture with $X_{A}=X_{B}=0.5$ as $F_{\mathrm{AB}}\left(\kappa_{A},\kappa_{B}\right)$, the sought after excess free energy is

(26) $$\Delta F_{\mathrm{exc}}\left(\kappa_{A},\kappa_{B}\right)=F_{\mathrm{AB}}\left(\kappa_{A},\kappa_{B}\right)-\left[F_{A}\left(\kappa_{A}\right)+F_{B}\left(\kappa_{B}\right)\right]/2.$$

Equation (26) leads to

(27) $$\frac{1}{N}\frac{\partial (\beta \Delta F_{\mathrm{exc}})}{\partial (\beta \kappa_{B})}=\frac{1}{N}\frac{\partial (\beta F_{\mathrm{AB}})}{\partial (\beta \kappa_{B})}-\frac{1}{2N}\frac{\partial (\beta F_{B})}{\partial (\beta \kappa_{B})},$$

since the number of bonds in the B chains is only one half of the total number of bonds per chain in the mixed system. Hence,

(28) $$\frac{1}{N}\frac{\partial (\beta \Delta F_{\mathrm{exc}})}{\partial (\beta \kappa_{B})}=\frac{1}{2}{\langle U_{\mathrm{bend}}^{B}\rangle }_{\mathrm{AB}}-\frac{1}{2}{\langle U_{\mathrm{bend}}^{B}\rangle }_{B}.$$

Here, $U_{\mathrm{bend}}^{B}$ is the bending energy per monomer of a B chain, ${\langle \ldots \rangle }_{\mathrm{AB}}$ denotes the thermal average in a 1:1 AB mixture, while ${\langle \ldots \rangle }_{B}$ refers to an average in a pure system of B chains. The desired free energy difference then is

(29) $$\frac{\beta \Delta F_{\mathrm{exc}}\left(\kappa_{A},\kappa_{B}\right)}{N}=\frac{1}{2}\int_{\beta \kappa_{A}}^{\beta \kappa_{B}}d\left(\beta \kappa_{B}^{\prime }\right)\left[{\langle U_{\mathrm{bend}}^{B}\left(\kappa_{B}^{\prime }\right)\rangle }_{\mathrm{AB}}-{\langle U_{\mathrm{bend}}^{B}\left(\kappa_{B}^{\prime }\right)\rangle }_{B}\right].$$

**Figure 12:**
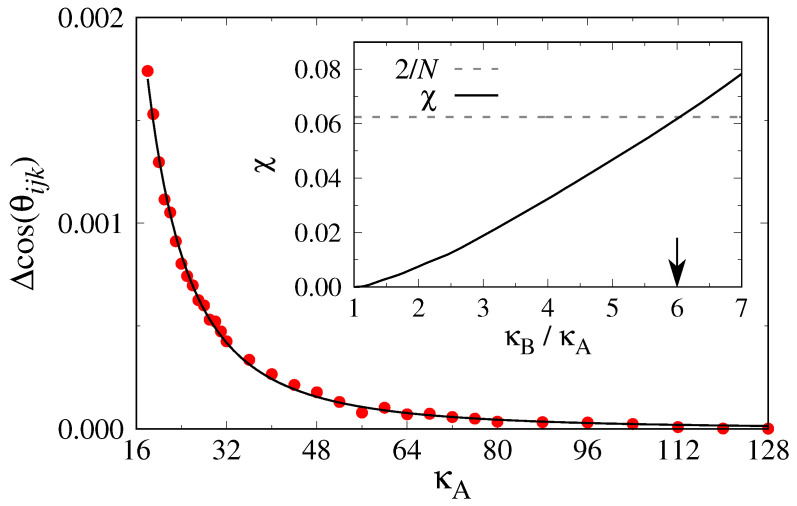
Difference in average bending angles $\langle \Delta \cos\left(\theta_{ijk}\right)\rangle$ versus $\kappa_{A}$ at constant monomer density $\rho =0.42\;\sigma^{-3}$ for the system $N_{A}=N_{B}=32$ and $\kappa_{B}=128$. The solid line is a guide to the eye only. The inset shows the resulting effective χ parameter versus $\kappa_{B}/\kappa_{A}$. The dashed horizontal line shows the mean field prediction for criticality in a symmetric mixture, $\chi_{c}=2/N$. The arrow indicates the ratio for which the two curves cross.

We have performed this thermodynamic integration for a system at $\rho =0.42\;\sigma^{-3}$ in a cubic box of size $L_{x}=L_{y}=L_{z}=64\;\sigma$ with N=111,232 monomeric units. We started the numerical integration in a state $\kappa_{A}=\kappa_{B}=128$, and then lowered the stiffness of the A component to smaller values (Figure 12). One sees that the integrand is extremely small if both chains have similar stiffness, but it rises steeply for large stiffness mismatches. The resulting effective χ parameter is shown in the inset of Figure 12. Criticality (according to mean-field theory) would be reached for $\kappa_{B}/\kappa_{A}\approx 6$, i.e., $\kappa_{A}^{c}\approx 21$. However, while the pressure $P\approx 0.29\;k_{B}T\sigma^{-3}$ for $\rho =0.42\;\sigma^{-3}$ and $\kappa_{A}=16$ clearly falls inside the i–n${}_{2}$ coexistence region, for $\kappa_{A}=20$, the critical pressure $P_{c}\approx 0.7\;k_{B}T\sigma^{-3}$ corresponds to $\rho_{c}=0.58\;\sigma^{-3}$; hence, this system at $\rho =0.42\;\sigma^{-3}$ is in the mixed nematic region. Since $\chi_{\mathrm{eff}}$ can be expected to increase with increasing density, we cannot estimate $\chi_{\mathrm{eff}}$ for the data shown in Figure 10, but the order of magnitude of $\chi_{\mathrm{eff}}$ expected on the basis of Figure 12 makes sense.

## 5. Conclusions

In this paper, we have considered binary mixtures of semiflexible polymers in a common good solvent. We have studied the case where both constituents have the same stiffness but differ in chain length and the case where both constituents have strictly the same chain length but have a large stiffness disparity. We have considered here only very simple models appropriate for lyotropic solutions, where nematic order is purely entropy-driven, and applied two complementary computational approaches: (i) Density Functional Theory, based on a tangent hard sphere model of chains where stiffness is controlled by a bond angle potential (Equation (1)), and (ii) Molecular Dynamics simulation, based on standard bead-spring models augmented with the same bond angle potential. We considered binary mixtures of chains with $N_{A}=16$ and $N_{B}=32$ monomeric units (of diameter σ and bond length between the units $\ell_{b}\approx \sigma$) with persistence length $\ell_{p}/\ell_{b}=32$, as well as mixtures with $N_{A}=16$ and $N_{B}=64$ with $\ell_{p}/\ell_{b}=128$, both at mole fraction $X_{B}=0.5$. It was found that the i–n coexistence regions are similarly narrow as for the corresponding pure systems. In the mixed nematic phase, the nematic order parameter $S_{A}$ of the shorter chains is always less than $S_{B}$ but larger than $S_{A}$ for the corresponding pure system.

The second case focused on chains of the same length, $N_{A}=N_{B}=32$, where the stiffer component is almost rod-like $\left(\ell_{p}^{B}/\ell_{b}=128\right)$, while the persistence length of the less stiff chain was much smaller $\left(16\leq \ell_{p}^{A}/\ell_{b}\leq 26\right)$. These systems also have a strong tendency for nematic order, but unlike the former cases exhibit relatively wide i–n two-phase regions. Again, the chains in nematic phases adapt to their environment; the less stiff chains are rather strongly stretched when they are dissolved in a stiffer matrix, whereas the inverse effect is much less pronounced. The stiffness mismatch between the two constituents has been shown to cause an unmixing tendency, which can be described by an effective Flory-Huggins χ parameter, although there are no attractive forces between monomeric units of different chains present whatsoever. We have computed this effective χ parameter for one density as function of $\kappa_{B}/\kappa_{A}$ (for $\kappa_{B}=128$) through thermodynamic integration, and have shown that mean-field theory based on this χ parameter would yield roughly the correct ratio $\kappa_{B}/\kappa_{A}$ for which this phase separation is actually observed. Using the random phase approximation, one predicts that critical correlations are very anisotropic in dilute and semi-dilute solutions: The correlation length parallel to the nematic director scales proportional to chain length *N*, while it only is proportional to $\sqrt{N}$ in the perpendicular direction. A thorough numerical investigation of this new type of critical behavior is very challenging, however, and must be left to the future. There has been great recent interest in the critical behavior of anisotropic systems in the Ising universality class [68,69], since the principal directions of order parameter correlations beyond mean-field theory may deviate from the expectations based on mean-field theory, and one can show that then the principle of “two-scale factor universality” [70] is violated.

This critical n${}_{1}$–n${}_{2}$ unmixing can be seen for an intermediate range of $\kappa_{B}/\kappa_{A}$ only: When $\kappa_{B}/\kappa_{A}$ is not large enough, the critical point would be at unphysically large monomer densities, where the nematic phase no longer exists at all (and smectic or crystal phases have taken over). On the other hand, when the ratio increases, a multi-critical point emerges (see Figure 3b), where the critical point touches the i–n coexistence region. For still larger $\kappa_{B}/\kappa_{A}$, it would be metastable, and the equilibrium phase diagram then exhibits a triple point (Figure 2a,b, Figure 3a and Figure 10a). For $\kappa_{B}=128$, DFT has predicted the multi-critical point where the phase diagram topology changes to occur for $\kappa_{A}^{M}=20.5$, while MD, rather, suggests $\kappa_{A}^{M}=18\pm 1$. We do not attribute this discrepancy to the minor differences between the chain models used by DFT and MD but, rather, hold responsible the approximations used by DFT, where only the orientation ω of a rod-like effective chain enters as local freedom, local density is not a variable, and the effective pairwise interaction $V_{\mathrm{excl}}\left(\omega ,\omega^{\prime }\right)$ used in Equation (10) is approximate. Nevertheless, the guidance provided by DFT to yield an overall picture of phase behavior and lead the interpretation of MD results is extremely valuable.

Unfortunately, we are not aware of real systems to which the present model calculations could be compared directly, although there have been studies of mixtures of two different semiflexible polymers in a common solvent. For example, Russo and Cao [44] obtained the phase diagram of poly(-γ-benzyl-α, L-glutamate) (PBLG) and Nylon 6 in m-cresol as a solvent. PBLG in various solvents has broadly been used as a model material of a liquid-crystal forming polymer in lyotropic solutions [1,2]. However, for the system of Reference [44], the case $N_{A}=N_{B}=N$ studied here has been out of focus, and, furthermore, the relevance of hydrogen-bond interactions was stressed, which makes the problem much more complicated.

## Data Availability

The data that support the findings of this study are available from the corresponding author upon reasonable request.

## Appendix A

**Table A1:** Partial densities and ratios $r_{1}$ and $r_{2}$ for a system with $N_{A}=N_{B}=32$, $\kappa_{A}=20$, and $\kappa_{B}=128$.

ρ	$\rho_{\mathrm{Aa}}$	$\rho_{\mathrm{Ab}}$	$\rho_{\mathrm{Bb}}$	$\rho_{\mathrm{Ba}}$	$r_{1}$	$r_{2}$
0.250	0.1574	0.0203	0.2641	0.0826	0.234	0.236
0.300	0.2358	0.0241	0.3109	0.0413	0.403	0.405
0.350	0.2987	0.0263	0.3650	0.0194	0.450	0.454
0.400	0.3552	0.0348	0.4077	0.0049	0.484	0.484
0.425	0.3793	0.0482	0.4181	0.0022	0.505	0.504
0.450	0.4018	0.0844	0.4013	0.0016	0.559	0.557
0.475	0.4033	0.0897	0.4134	0.0407	0.528	0.529
0.500	0.4159	0.0873	0.4421	0.0546	0.504	0.505
0.550	0.4657	0.0805	0.4997	0.0520	0.498	0.495
0.600	0.5325	0.0517	0.5859	0.0328	0.483	0.484

**Table A2:** Partial densities and ratios $r_{1}$ and $r_{2}$ for a system with $N_{A}=N_{B}=32$, $\kappa_{A}=16$, and $\kappa_{B}=128$.

ρ	$\rho_{\mathrm{Aa}}$	$\rho_{\mathrm{Ab}}$	$\rho_{\mathrm{Bb}}$	$\rho_{\mathrm{Ba}}$	$r_{1}$	$r_{2}$
0.250	0.1606	0.0405	0.2380	0.0790	0.289	0.296
0.275	0.2012	0.0473	0.2560	0.0558	0.408	0.414
0.300	0.2377	0.0582	0.2681	0.0387	0.485	0.489
0.325	0.2710	0.0731	0.2771	0.0250	0.545	0.548
0.350	0.2991	0.0963	0.2752	0.0159	0.614	0.612
0.600	0.3867	0.2629	0.3418	0.2030	0.699	0.700
0.615	0.4324	0.1927	0.4372	0.1671	0.520	0.521
0.630	0.4680	0.1714	0.4789	0.1438	0.511	0.516
0.650	0.5043	0.1494	0.5224	0.1226	0.506	0.505
0.670	0.5587	0.1033	0.5955	0.0857	0.489	0.491
0.690	0.5931	0.0598	0.6684	0.0654	0.464	0.465
